# Detection of Viral Hemorrhagic Septicemia virus (VHSV) from the leech *Myzobdella lugubris *Leidy, 1851

**DOI:** 10.1186/1756-3305-2-45

**Published:** 2009-09-28

**Authors:** Mohamed Faisal, Carolyn A Schulz

**Affiliations:** 1 Department of Fisheries and Wildlife, Michigan State University, S-112 Plant Biology Building, East Lansing, MI, 48824, USA; 2 Department of Pathobiology and Diagnostic Investigation, Michigan State University, 4125 Beaumont Road, Lansing, MI, 48910, USA

## Abstract

The leech *Myzobdella lugubris *is widespread in the Lake Erie Watershed, especially Lake St. Clair. However, its role in pathogen transmission is not fully understood. In this same watershed, several widespread fish mortalities associated with the Viral Hemorrhagic Septicemia virus (VHSV) were recorded. Viral Hemorrhagic Septicemia is an emerging disease in the Great Lakes Basin that is deadly to the fish population, yet little is known about its mode of transmission. To assess the potential role of *M. lugubris *in VHSV transmission, leeches were collected from Lake St. Clair and Lake Erie and pooled into samples of five. Cell culture and reverse transcriptase polymerase chain reaction (RT-PCR) were used to determine the presence of the virus and its identity. Results showed that 57 of the 91 pooled leech samples were positive by cell culture for VHSV and 66 of the 91 pooled leech samples were positive by RT-PCR for the VHSV. Two representative virus isolates were sequenced for further genetic confirmation and genotype classification. VHSV detected within *M. lugubris *was homologous to the Great Lakes strain of VHSV genotype IVb. This is the first record of the VHSV being detected from within a leech, specifically *M. lugubris*, and suggests the potential of *M. lugubris *being involved in VHSV transmission.

## Findings

The Viral Hemorrhagic Septicemia Virus (VHSV), genotype IVb, is a recent invader to the Great Lakes Basin (GLB) and has been associated with mortalities in a number of freshwater fish species [1-4]. These recent widespread mortality events in the GLB have raised questions concerning potential routes of virus transmission.

Certain leech species have been incriminated as potential vectors for fish viruses, such as, *Piscicola salmositica *for Infectious Hematopoietic Necrosis Virus in the sockeye salmon, *Oncorhynchus nerka *Walbaum [5] and *P. geometra *for Spring Viraemia of Carp Virus in the case of the common carp, *Cyprinus carpio *Linnaeus [6]. In a previous study, the leech population in Lake St. Clair, Michigan was found to be dominated by *Myzobdella lugubris *Leidy, 1851 (Rhynchobdellida: Piscicolida) [Schulz CA, Thomas MV, Fitzgerald S, Faisal M: **Leeches (Annelida: Hirudinea) Parasitizing Fish of Lake St. Clair, Michigan**. *Submitted*]. *Myzobdella lugubris *is an intermittent, haematophagous feeder, with an extraordinary wide host range [7-9] and therefore it is a good candidate leech to contribute to pathogen spread among susceptible host species. In this study we collected attached *M. lugubris *from fish collected from two sites in the Lake Erie watershed, where VHSV Type IVb was first isolated, and subsequent fish mortalities have taken place over the last few years [1].

**Figure 1:**
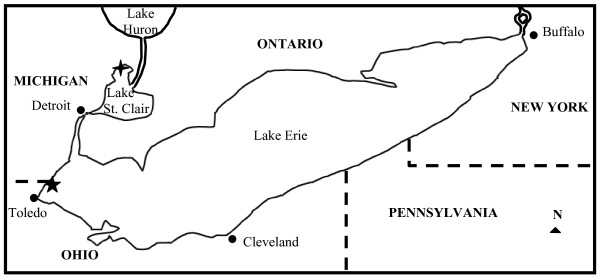
**The Lake Erie Watershed is connected in the east to Lake Ontario by the Welland canal and in the west to Lake Huron via the Detroit River, Lake St. Clair, and the St. Clair River**. The four-pointed black star denotes the sampling location of the Michigan Department of Natural Resources trap nets (42°37'54.60"N, 82°45'54.60"W) in Anchor Bay, Lake St. Clair. The five-pointed black star denotes the commercial fishing trap nets (41°46'00.74"N, 83°24'58.09"W) in Lake Erie from which leeches were collected during this study.

Leeches were collected on five separate dates, within the months of May and June 2008, from a site in Anchor Bay, Lake St. Clair (42°37'54.60"N, 82°45'54.60"W) and a site in western Lake Erie (41°46'00.74"N, 83°24'58.09"W) (Figure 1). Fish species from which *M. lugubris *was collected included the channel catfish (*Ictalurus punctatus *Rafinesque), freshwater drum (*Aplodinotus grunniens *R.), rock bass (*Ambloplites rupestris *R.), yellow perch (*Perca flavescens *Mitchill), and walleye (*Sander vitreus *M). Due to the intermittent feeding nature of *Myzobdella lugubris*, samples collected during this study were not separated according by fish species mentioned above or by specific location.

Detached leeches were tentatively identified as *M. lugubris *in the field and were stored in one liter bottles containing lake water. Overall, 456 leeches were removed and divided into 91 pools of ~five leeches. Leeches remained alive until returned to the laboratory, where their identity was confirmed as *M. lugubris *according to the accepted morphological criteria [9,10]. Each leech was briefly immersed into absolute ethanol for surface disinfection, rinsed several times in sterile water, and then sectioned into ~100 μg pieces. Samples were homogenized using a Biomaster Stomacher (Wolf Laboratories Ltd, Pocklington, York, UK) at the high speed setting for 2 min and then diluted with Earle's salt-based minimal essential medium (MEM, Invitrogen, Carlsbad, CA) to produce a 1:4 dilution (w/v) of original tissues. Homogenized leech contents were removed with a sterile transfer pipette, dispensed into a sterile 15 ml centrifuge tube, and centrifuged at 5500 rcf for 20 min in the IEC Multi RF Centrifuge (Thermo Fisher Scientific, Pittsburgh, PA). Supernatants were immediately used for virus isolation.

Virus isolation was performed according to the standard protocols detailed in the American Fisheries Society Blue Book [11] and the OIE [12], using the *Epithelioma papulosum cyprinii *(EPC) cell line [13]. Inoculated 96-well plates containing EPC cells grown with MEM (5% fetal bovine serum) were incubated at 15°C for 7 days, and were observed for the formation of cytopathic effects (CPE). Second and third blind passages were performed and assessed for the presence of VHSV.

Thirteen of the 91 pooled samples of leech homogenates caused CPE on EPC in the form of focal areas of rounded, refractile cells which progressed to full lysis of the cell monolayer. When a second passage was performed on negative samples, four additional samples exhibited CPE. A third passage raised the number of positive samples to 57 out of 91 pools. It is possible that the virus was present in higher titers in the samples which were positive in the first passage of cell culture.

**Figure 2:**
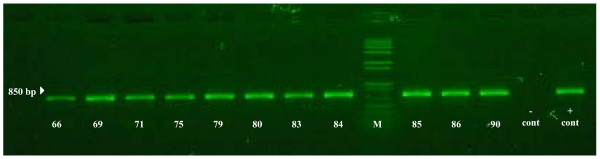
**Agarose gel showing the bands from RT-PCR, used for the detection of VHSV (811 base pair)**. Pooled leech samples (#66, 69, 71, 75, 79, 80, 83-86, and 90) are representative VHSV-positive samples. The marker (M) used was 1.0 kb plus (Invitrogen).

Reverse transcriptase polymerase chain reaction (RT-PCR) was then performed on all positive and negative third passage pooled leech samples. Total RNA was extracted from inoculated cells using a QIAamp^® ^Viral RNA Mini Kit. Reverse transcription was accomplished by a two-step protocol using the Affinity Script Multiple Temperature Reverse Transcriptase RT-PCR™. The primer set used in this assay was recommended by the Office de International Epizootics for detection of a 811 base pair sequence of the VHSV nucleocapsid (N) gene: 5'-GGG GAC CCC AGA CTG T-3' (forward primer) and 5'-TCT CTG TCA CCT TGA TCC-3' (reverse primer) [12]. Amplicons of 811 base pairs were amplified in 66 out of the 91 samples (Figure 2), including 56 out of the 57 cell culture positive samples, as well as ten additional samples that never formed CPE on EPC. Also, sample #65, which produced CPE on EPC, was negative by RT-PCR. After initial detection of VHSV Type IVb via RT-PCR, additional confirmation of positive samples was performed by the United States Department of Agriculture National Veterinary Services Laboratory in Ames, Iowa.

The RT-PCR products of two representative VHSV-positive samples were purified with the Promega Wizard^® ^SV Gel and PCR Clean-up System and were then submitted to the MSU Research Technology Support Facility. The two sequences were aligned by BL2SEQ [14] and the aligned contig was used for multiple alignments performed by ClustalW [15]. The phylogenetic analysis of the VHSV leech strain with 19 nucleoprotein encoding genes from other species of rhabdovirus was done by using bootstrap test of phylogeny in MEGA 4 [16]. The Neighbor-Joining algorithm was chosen to create the phylogenetic dendrogram containing 1000 bootstrap samplings.

Sequencing of the two leech isolates produced a 780 base pair sequence (GenBank:1227728) that was identical to the VHSV IVb-MI03 strain, the index strain of the Great Lakes VHSV (GenBank:DQ427105).

Our findings shed light on the potential role leeches may play in VHSV transmission. While this study does not confirm that *Myzobdella lugubris *does indeed transmit the virus to susceptible hosts, this is the first time that VHSV (of any genotype) has been isolated from leeches, or other invertebrates. *Myzobdella lugubris *is an intermittent, generalist species; therefore the detection of VHSV within *M. lugubris *may pose a threat to VHSV-susceptible host species, not only in the Great Lakes basin, but also in other watersheds to which infected *M. lugubris *may be transferred.

## Competing interests

The authors declare that they have no competing interests.

## Authors' contributions

CS conducted field collection and all laboratory assays, while CS and MF designed the study and drafted the manuscript. Both authors reviewed and approved of the final manuscript.
